# Semi-Quantitative ΔCt Thresholds for Bacteriuria and Pre-Analytic Drivers of PCR-Culture Discordance in Complicated UTI: An Analysis of NCT06996301

**DOI:** 10.3390/diagnostics15232959

**Published:** 2025-11-21

**Authors:** Moustafa Kardjadj, Itoe P. Priestly, Roel Chavez, DeAndre Derrick, Thomas K. Huard

**Affiliations:** 1 dicentra, Toronto, ON M4W 3E2, Canada; 2 Soft Cell Laboratories, Saint George, UT 84770, USA; 3 Doc Lab Inc., Hillsboro, OR 97006, USA; 4 MED-US Consulting, LLC., Austin, TX 78734, USA

**Keywords:** multiplex PCR, urine culture, ΔCt, semi-quantitative PCR, pre-analytic variability, complicated urinary tract infection, diagnostic concordance, time-to-processing

## Abstract

**Background**: Quantitative urine culture (CFU/mL) remains the reference standard for diagnosing urinary tract infections (UTIs) but is limited by delayed turnaround times and sensitivity to pre-analytic factors. Multiplex PCR panels offer rapid detection; however, standardized mappings between molecular signals and viable bacterial burdens are insufficiently defined. We used the multicenter NCT06996301 paired dataset to evaluate the analytical validity (AV), clinical validity (CV), and pre-analytic robustness of ΔCt (Ct_target − IC_Ct) as a semi-quantitative indicator of bacterial load. **Methods**: We analyzed 1027 paired PCR and quantitative urine culture specimens from six sites. The primary molecular predictor was ΔCt (Ct_target − IC_Ct). Species-level Spearman and Pearson correlations, species-specific linear mixed-effects calibration models (log_10_CFU ~ ΔCt + (1|site)), and ROC analyses were performed for the taxa meeting pre-specified sample thresholds. A pooled multilevel model assessed the collection method and time-to-processing (hours) effects (log_10_CFU ~ ΔCt × collection_method + ΔCt × time_to_processing_h + (1|site) + (1|run)). AV was assessed via reproducibility, internal control normalization, and site run variance. CV was assessed by ΔCt calibration and discrimination. Clinical utility (CU) was contextualized using outcomes from the parent randomized trial. **Results**: PCR positivity exceeded culture positivity across all sites (PCR ~82–88% vs. culture ~66–70%); this excess likely reflects a combination of molecular detection of non-viable DNA, detection of fastidious taxa less readily recovered by culture, and pre-analytic effects. For six common uropathogens (*n* = 90 pairs/species), ΔCt correlated strongly with log_10_CFU (Spearman ρ = −0.64 to −0.75; Pearson r = −0.75 to −0.83). Species-specific mixed models yielded slopes of −0.746 to −0.922 log_10_CFU per ΔCt unit (all *p* < 0.001), indicating that each 1 unit ΔCt change corresponds to a ~5.6–8.4-fold CFU difference. ROC AUCs for ΔCt discrimination were 0.78–0.84 (interpreted as good discrimination, i.e., ΔCt meaningfully improves the clinician’s probability estimate of a high CFU but does not perfectly classify every specimen). The collection method (catheter vs. clean-catch) did not materially modify the ΔCt→CFU relationship, whereas the processing delay was associated with reduced recovered CFU (~0.048 log_10_CFU lost per hour) and a significant ΔCt × time interaction, consistent with time-dependent viability loss driving the PCR^+^/culture^−^ discordance. **Conclusions**: ΔCt from the DOC Lab UTM 2.0 panel shows a reproducible, analytically valid semi-quantitative measure of urinary bacterial load. Laboratories can derive assay- and workflow-specific ΔCt cut points for semi-quantitative reporting, but thresholds must be validated prospectively and paired with operational controls for specimen handling.

## 1. Introduction

Quantitative urine culture (colony-forming unit “CFU”/mL with isolate-level phenotypic antibiotic susceptibility testing, “AST”) remains the clinical standard for diagnosing urinary tract infections (UTIs) and for guiding antimicrobial therapy, because culture establishes viability and enables phenotypic susceptibility testing. Nevertheless, culture has recognized limitations; delayed turnaround, reduced sensitivity after prior antibiotics, and under-reporting of polymicrobial or fastidious infections, which impede timely, targeted therapy [1,2,3]. Multiplex molecular diagnostics (real-time PCR panels) provide rapid, broad-spectrum detection of uropathogens and key resistance markers, and therefore have the potential to shorten time-to-effective therapy and improve clinical outcome [3,4,5,6,7,8].

A key barrier to clinical implementation is that most PCR reports are binary (“detected/not detected”), which leaves clinicians without actionable information about organism burden or the probability that a positive result reflects true infection, rather than colonization or environmental contamination. This lack of semi-quantitative mapping hinders common clinical decisions in cUTI: for example, whether to start empiric broad-spectrum therapy, to wait for culture and AST, or to perform a rapid stewardship review, because the clinician cannot reliably translate a positive PCR into an expected culture burden or risk of phenotypic resistance [2,3,4]. It also constitutes a gap in the demonstrable analytical validity (AV) and clinical validity (CV): the quantitative relationship between the molecular signal and viable bacterial burden must be clear, assay-specific, and reproducible [2].

Despite this promise, two related barriers have slowed the routine clinical adoption of PCR-based panels for management decisions in a complicated UTI (cUTI). First, there are no universally standardized, clinically validated mappings between molecular signals (cycle threshold (Ct)/quantification cycle (Cq)) and bacterial burdens (CFU/mL); reported Ct↔CFU relationships are assay- and organism-specific, varying with specimen handling and platform [2,3,4]. Second, pre-analytic variability (including collection method, transport conditions, time to processing, and site/run effects) can alter both culture viability and molecular signal, thereby confounding PCR↔culture concordance and clinical interpretation; without per-sample internal-control reporting and clear QC rules, semi-quantitative results cannot be trusted for therapeutic decisions [9,10]. Finally, payer and regulatory frameworks increasingly require evidence of assay-specific analytic and clinical validity (including measures of reproducibility, calibration, and operational stability) before routine coverage for syndromic molecular panels can be approved; assay-specific ΔCt thresholds and QC dashboards are directly relevant to meeting those expectations [2,9,11].

The randomized, investigator-blinded NCT06996301 dataset [12,13,14], with paired PCR and quantitative culture on each urine specimen, per-sample internal-control Ct, detailed collection/processing metadata, and clinical outcomes, provides a rich platform to address both gaps. Our central hypothesis is that, within a validated operational workflow, target- and species-specific ΔCt (Ct target − IC_Ct) calibrations can reproducibly map to clinically meaningful culture thresholds (e.g., ≥10^3^, ≥10^4^, ≥10^5^ CFU/mL), and that explicit QC metadata (IC_Ct ranges, time-to-processing) will materially improve the interpretability and generalizability of these mappings. The specific aims of this ad hoc analysis are the following: (1) derive per-target ΔCt (Ct target − IC_Ct “Internal Control”)↔log_10_(CFU/mL) calibration curves and produce clinically actionable ΔCt thresholds, mapping to established culture cutoffs (≥10^3^, ≥10^4^, ≥10^5^ CFU/mL) to satisfy analytical validity expectations; (2) quantify ΔCt discriminatory performance relative to clinical culture thresholds, and (3) quantify pre-analytic robustness by evaluating internal-control Ct distributions, site/run variability, collection method (catheter vs. clean catch), and time from collection to processing as drivers of PCR–culture discordance. By generating target-level Ct thresholds together with site-level QC dashboards and reporting conventions, we aim to produce the concrete, assay-specific evidence (analytic calibration, reproducibility metrics, and operational boundaries) that clinicians, laboratory directors, and payers require to use semi-quantitative PCR outputs in real-world cUTI management.

## 2. Methods

### 2.1. Study Design (NCT06996301)

NCT06996301 was a multicenter, randomized, parallel, investigator-blinded clinical trial conducted at six geographically distinct clinical sites (physician office laboratories, “POLs”) to evaluate the clinical utility of a multiplex PCR panel vs. conventional quantitative urine culture and susceptibility testing (C&S) for the management of complicated urinary tract infections (cUTI) in adults. The trial design and primary results have been published previously [12,13,14]. NCT06996301 was registered at the clinicalTrial.gov registry (https://clinicaltrials.gov/study/NCT06996301?term=NCT06996301&rank=1, accessed on 16 November 2025). The trial and the present ad hoc analysis were approved by an independent institutional review board (Advarra IRB, Pro00071764; approval date: 22 May 2023). Trial conduct and data management were overseen by an independent contract research organization (dicentra CRO, Study ID: 22-UPHUV-01). This ad hoc analytical validation supplements the parent trial by evaluating the semi-quantitative validity of ΔCt (Ct_target − IC_Ct).

**Figure 1:**
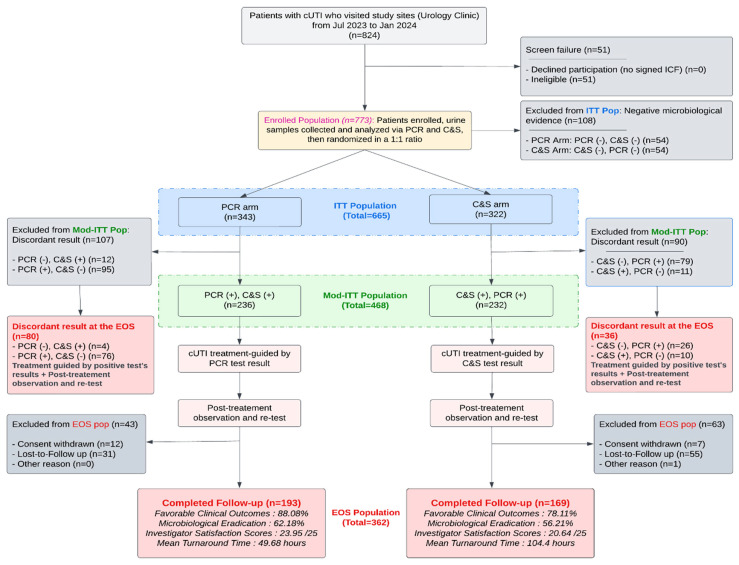
The CONSORT flow diagram detailing the schedule of events and the findings. All enrolled participants (*n* = 773) underwent both multiplex PCR and conventional culture and susceptibility (C&S) testing at the baseline, prior to randomization into one of two study arms. Treating investigators were permitted to make clinical management decisions based solely on the diagnostic results from their assigned arm and remained blinded to the comparator test results until the end-of-study (EOS). The favorable clinical outcome (FCO) was defined as the resolution of at least one baseline cUTI symptom without the emergence of new symptoms, as assessed by the site investigator. Microbiological eradication was defined as the absence at EOS of all baseline uropathogens by quantitative culture.

A CONSORT flow diagram describing enrollment, allocation, and specimen processing is provided in Figure 1.

### 2.2. Source Data and Analytic Population

An ad hoc extract from the trial database was used to generate site-level pre-analytic and assay quality control (QC) summaries and all analytic datasets for this manuscript. The working dataset included 1027 clinical urine samples (intention-to-treat population n = 665 and end-of-study population *n* = 362), where each row corresponds to a single urine specimen with paired molecular and culture results.

Available sample-level metadata included study site, sample identifier, clinical symptoms, collection method (clean catch vs. catheter), date/time of collection, date/time of laboratory receipt, calculated time to processing (hours), raw target Ct values for up to 28 uropathogen targets and 16 resistance gene targets, internal control Ct (IC_Ct), PCR run and failure flags (including inhibition indicator, IC_flag), quantitative culture result (CFU/mL reported as log_10_CFU), isolate-linked MIC (minimum inhibitory concentration) values (AST_MIC, numeric) and categorical AST interpretation (AST_SIR: S/I/R), prior antibiotic exposure (none in the last 48 h, as per the trial’s eligibility criteria), randomized assignment (PCR-guided arm vs. culture-guided arm), and recorded clinical outcomes. Data access and secondary analyses were performed under the IRB allowances of the parent protocol.

### 2.3. Laboratory Essays and Key Variables

Molecular testing was performed using the DOC Lab UTM 2.0 multiplex PCR panel, which reports qualitative detection for 28 target species and 16 classes of resistance determinants, together with per-sample internal control amplification. Quantitative cultures were reported as CFU/mL and converted to log_10_CFU for analysis. Detailed laboratory procedures, platform performance characteristics, and quality-control workflows are described in the trial methods [12,13,14] and are summarized in the Methods from Supplementary Materials.

The primary molecular predictor used throughout the analysis was ΔCt = Ct_target − IC_Ct, which normalizes the target Ct to the sample internal control and reduces sample-level extraction/amplification variability. Secondary molecular predictors included the raw target Ct (sensitivity analyses). The pre-analytic covariates that were available and used in multivariable models included collection method (clean-catch vs. catheter) and time from collection to processing in hours (time_to_processing_h). PCR run identifiers and site identifiers were retained for clustering in multilevel models. PCR inhibition/run-failure flags were retained and used for exclusion/sensitivity checks.

### 2.4. Data Cleaning and Inclusion Criteria

Each row of the working dataset corresponds to one clinical urine specimen with paired PCR and culture results. Samples flagged as PCR run failures or with a flagged inhibition (IC_flag) were summarized separately; these were excluded from the primary calibration and ROC analyses to minimize technical artifacts.

Species-level analyses were restricted by prespecified minimum sample sizes to ensure stable estimates: species required *N* ≥ 90 matched PCR–culture pairs to be included in the correlation and species-specific mixed-effects calibration analyses, and *N* ≥ 90 matched pairs with class variation (at least one observation above and below the threshold) to be included in ROC analyses. To evaluate the robustness of these thresholds, an additional sensitivity analysis, including all species with *N* ≥ 50 matched pairs, was conducted.

Non-detected targets (no amplification) and those not meeting the matched PCR–culture pairs threshold were treated as censored observations. In the primary analysis, non-detects were assigned the manufacturer’s limit-of-detection (LOD) Ct to retain continuous scaling. A sensitivity analysis was conducted, excluding non-detects; the results were consistent with the main analysis and therefore were not reported.

### 2.5. Statistical Analysis

All statistical analyses were performed in R (version 4.5.2). Primary packages included lme4 and lmerTest for mixed models, pROC for ROC analyses, MuMIn for R^2^ calculations, broom for tidy model outputs, and base and recommended R libraries for data manipulation and plotting. Two-sided hypothesis tests were used throughout with a nominal, α = 0.05.

#### 2.5.1. Descriptive Summaries and Site QC

The site-level quality metrics computed for each POL included the number of samples, percent culture positive, percent PCR positive (any target), percent discordant results (PCR^+^/Culture^−^ and PCR^−^/Culture^+^), median log_10_CFU among culture positives, median target Ct among PCR positives, median IC_Ct, percent inhibited, percent PCR run failures, and percent of samples collected by catheter. IC_Ct distributions and inhibition/run-failure rates were inspected to assess cross-site extraction and amplification consistency.

#### 2.5.2. Ct→CFU Correlation (Per-Species), Calibration, and ROC Analyses

##### Correlation Analyses

For each species meeting the *N* ≥ 90 requirement, monotonic relationships between ΔCt and log_10_CFU were assessed with the Spearman rank correlation (ρ) and linear relationships with the Pearson correlation (r). Two-sided *p*-values are reported for all correlation tests.

The *N* ≥ 90 inclusion threshold was pre-specified to ensure stable parameter estimation and guard against overfitting in per-species regression and ROC models. This cutoff was derived empirically, based on simulation and the prior literature on quantitative microbiology [7], which shows that ΔCt-CFU relationships stabilize when ≥80–100 paired observations are available per species, particularly under a heteroscedastic measurement error. A sensitivity analysis, including additional species strata with *n* ≥ 50, was conducted to confirm that results were robust to this threshold (see Section 2.5.3).

##### Species-Specific Calibration Models

Species-specific linear mixed-effects models were fit to quantify the average change in log_10_CFU per 1 unit change in ΔCt, while accounting for clustering by site. The primary model specification was as follows:

$${\log_{10}\mathrm{CFU}}_{ij}=\beta_{0}+\beta_{1}\;\Delta {\mathrm{Ct}}_{ij}+u_{{\mathrm{site}}_{j}}+\varepsilon_{ij},$$

where $u_{{\mathrm{site}}_{j}}∼N(0,\sigma_{\mathrm{site}}^{2})$ is a random intercept for site and $\varepsilon_{ij}∼N(0,\sigma^{2})$ is the residual error. Models were fit by the restricted maximum likelihood (REML). For each species, we report the slope estimate (*β*_1_), standard error, t-statistic, two-sided *p*-value, 95% Wald confidence interval, and marginal and conditional R^2^ (variance explained by fixed effects and by fixed + random effects, respectively).

To evaluate the necessity of the random site effect, model selection was performed by comparing Akaike Information Criterion (AIC) and Bayesian Information Criterion (BIC) values between the mixed model [log_10_CFU ~ ΔCt + (1|site)] and a simpler fixed-effects model [log_10_CFU ~ ΔCt]. In most species, the random-effect variance was near-zero, indicating limited between-site heterogeneity; these were reported as “singular” fits. For transparency, fixed-effects sensitivity models were refitted in such cases to confirm that slope estimates were consistent across specifications. The inclusion of a random intercept was retained for consistency with the broader hierarchical structure of the trial dataset and to maintain comparability across species-level and pooled analyses.

##### Discriminatory (ROC) Analyses

We evaluated the ΔCt clinical validity to discriminate the culture burden at clinically relevant cutoffs. ROC curves and area under the curve (AUC) with 95% confidence intervals (DeLong method) were computed for species meeting the *N* ≥ 90 rule and where both binary outcome classes were present for the tested threshold. Where class variation was absent (all observations for a species fell on one side of a threshold), ROC analysis was not performed and the species–threshold pair was skipped.

Operational cut-points were derived using the Youden index (maximizing sensitivity + specificity − 1). To quantify uncertainty around diagnostic accuracy metrics, bootstrap resampling (2000 replicates) was used to estimate 95% confidence intervals for sensitivity, specificity, positive predictive value, and negative predictive value.

In addition, the pooled multilevel model incorporated interaction terms (ΔCt × collection_method and ΔCt × time_to_processing_h) to formally test whether pre-analytic variables modified the relationship between molecular signal and viable bacterial load (Section 2.5.4). The ΔCt × collection_method term assessed whether the sample type (catheter vs. clean-catch) affected the calibration slope, while the ΔCt × time_to_processing_h term tested whether delayed specimen processing attenuated the culture yield for a given ΔCt value. These interaction terms were hypothesis-driven and grounded in the established literature on pre-analytic variability in molecular microbiology.

#### 2.5.3. Sensitivity Analysis for Ct→CFU Correlation, Calibration, and ROC Analyses

To assess the robustness of the primary Ct→CFU findings, a pre-specified sensitivity analysis was performed, including eight species with at least 50 matched PCR–culture pairs (*E. coli*, *Klebsiella* spp., *Enterococcus* spp., *Pseudomonas* spp., *Proteus* spp., coagulase-negative *Staphylococcus*, *Serratia* spp., and *Enterobacter* spp.). The lower inclusion threshold (*n* ≥ 50) allowed for the evaluation of additional clinically relevant species while maintaining adequate power for model stability.

Correlation analyses used the Spearman (ρ) and Pearson (r) coefficients to quantify monotonic and linear relationships between ΔCt and log_10_CFU, with two-sided *p*-values reported.

Mixed-effects models (log_10_CFU ~ ΔCt + IC_Ct + collection_method + prior_abx + (1|site)) were fitted using REML to estimate ΔCt slopes (α_1_). Model fit (AIC/BIC) supported retaining the site random effect; however, OLS sensitivity checks confirmed slope stability when site variance was negligible. Bootstrap percentile 95% CIs (2000 replicates) were computed for α_1_ using bootMer().

ROC analyses assessed ΔCt discrimination of the high culture burden (≥10^5^ CFU/mL), using −ΔCt as a predictor. AUCs (95% CI, DeLong method), Youden cut points, sensitivity, specificity, and prevalence were estimated with bootstrap resampling (2000 replicates) for interval precision.

#### 2.5.4. Multilevel Modeling of Pre-Analytic Drivers

To assess the influence of pre-analytic factors and ΔCt-CFU relationships, a pooled multilevel linear mixed-effects model was fitted to sample–target pairs with the following specification:

$$\begin{array}{cc} & {{log}_{10}\mathrm{CFU}}_{ijk}=\gamma_{0}+\gamma_{1}\;\Delta {\mathrm{Ct}}_{ijk}+\gamma_{2}\;{\mathrm{collection}\_\mathrm{method}}_{ijk}+\gamma_{3}\;{\mathrm{time}\_\mathrm{to}\_\mathrm{processing}\_h}_{ijk}\\ & +\gamma_{4}\;(\Delta \mathrm{Ct}\times \mathrm{collection}\_\mathrm{method}{)}_{ijk}+\gamma_{5}\;(\Delta \mathrm{Ct}\times \mathrm{time}\_\mathrm{to}\_\mathrm{processing}\_h{)}_{ijk}+u_{{\mathrm{site}}_{j}}+u_{{\mathrm{run}}_{k}}+\varepsilon_{ijk}, \end{array}$$

where $u_{\mathrm{site}}$ and $u_{\mathrm{run}}$ are random intercepts for the site and PCR run, respectively, and ε is the residual error. This model evaluates whether specimen handling modifies the ΔCt calibration slope, fulfilling the clinical utility requirements for assessing real-world operational stability. Models were fit by REML; when likelihood-ratio testing or model comparison was required, models were refit by the maximum likelihood (ML). Fixed-effect significance tests used Satterthwaite approximations for denominator degrees of freedom (lmerTest). Covariates were centered as being appropriate for interaction interpretation. Collinearity diagnostics (variance inflation factors) and residual diagnostics (normality, heteroscedasticity) were inspected. If inclusion of both site and run random effects resulted in singular fits, alternative parameterizations (e.g., single random intercept, random slopes, or robust standard errors) were evaluated and justified; the model presented in the Results represents the chosen parameterization.

## 3. Results

A total of 1027 urine specimens (intention-to-treat population *n* = 665 and end-of-study population *n* = 362) collected across six clinical sites were included in the analysis: Augusta (*n* = 205), Albany (*n* = 192), Norman (*n* = 224), Phoenix (*n* = 157), Silicon Valley (*n* = 29), and Southeastern (*n* = 220). All analyses presented below use the paired PCR and quantitative culture results for these specimens.

### 3.1. Site QC and IC Distributions

Site-level quality-control metrics are summarized in Table 1. At every site, the proportion of PCR-positive specimens exceeded the proportion of culture-positive specimens; site-level PCR positivity ranged from 82% to 88%, while culture positivity ranged from 66% to 70% (Table 1). The PCR^+^/culture^−^ fraction was consistently larger than the PCR^−^/culture^+^ fraction across sites (site-level PCR^+^/C^−^ ≈ 21–24% vs. PCR^−^/C^+^ ≈ 5–7%), yielding cohort-level discordance rates of 24.0% and 6.0%, respectively. Median culture burdens (log_10_CFU) among culture-positive specimens varied modestly by site (range 4.85–5.63), with Silicon Valley showing the highest median (5.63) but a small sample size (*n* = 29). Internal-control Ct (IC_Ct) medians were tightly clustered across sites (25.02–26.22) and the reported IC Ct min–max windows were comparable (10–31/33), consistent with broad uniform extraction and amplification performance. All sites adhered to standardized internal QC acceptance criteria, based on manufacturer specifications. Each PCR run included internal amplification controls and external positive controls for each target organism derived from authenticated ATCC strains, ensuring run validity and assay integrity across instruments and locations.

Percent inhibition and PCR-run failure rates were low across all sites (inhibition 0.64–1.95%; PCR failures 0–1.75%), confirming that inhibition and run failure were rare events in this dataset.

The observed PCR^+^/culture^−^ discordance rate (24%) represents the overall cohort-level estimate. Detailed breakdowns by pathogen species, time-to-processing interval, and specimen type have been reported previously [13].

Catheter-collected specimens accounted for 80% (*n* = 179) of samples from Norman, while all other sites reported 0%, reflecting differences in collection practices between facilities.

### 3.2. Ct→CFU Correlation (Per-Species), Calibration, and ROC Analyses

#### 3.2.1. ΔCt Correlations with Quantitative Culture

Species-level correlations between normalized molecular signal and viable bacterial burden are shown in Table 2. For the six species with *N* = 90 matched PCR–culture pairs (*E. coli*, *Klebsiella* spp., *Enterococcus* spp., *Pseudomonas* spp., *Proteus* spp., and coagulase-negative *Staphylococcus*), ΔCt (Ct_target − IC_Ct) exhibited strong, statistically significant inverse associations with log_10_CFU. Spearman rank correlations (ρ) ranged from −0.641 to −0.751 and Pearson correlations (r) ranged from −0.751 to −0.829; all reported *p*-values were <1 × 10^−11^ (Table 2). These findings indicate a robust monotonic and approximately linear relationship between normalized PCR signal and culture-quantified bacterial load across the species examined. This shows that lower ΔCt values (corresponding to higher molecular signal) are consistently associated with a higher viable bacterial burden across all six taxa.

A subset of targets in the DOC Lab UTM 2.0 panel did not meet the pre-specified minimum matched-pairs rule for species-level Ct↔CFU calibration and ROC modeling, and therefore were censored. Importantly, many of these low-count strata correspond to organisms that have long been recognized as fastidious or difficult-to-culture uropathogens, rather than to technical failures of the molecular assay.

#### 3.2.2. Species-Specific Mixed-Effects Calibration

**Figure 2:**
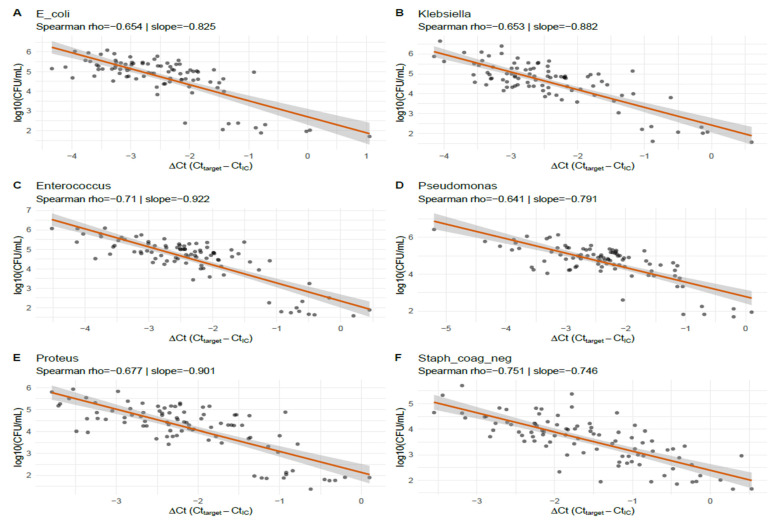
Species-specific ΔCt↔log_10_(CFU/mL) scatter panels with mixed-effects fit lines for six common uropathogens. Scatter plots show observed specimen-level log_10_CFU (y-axis) vs. ΔCt (Ct_target − IC_Ct, x-axis) for *E. coli* (**A**), *Klebsiella* spp. (**B**), *Enterococcus* spp. (**C**), *Pseudomonas* spp. (**D**), *Proteus* spp. (**E**), and coagulase-negative *Staphylococcus* (**F**). Each panel reports the Spearman rank correlation (ρ) and the species-specific mixed-effects slope (change in log_10_CFU per 1 unit ΔCt). The fitted lines and point cloud illustrate the strong, inverse ΔCt→CFU relationship across species.

Species-specific linear mixed-effects calibration models (log_10_CFU ~ ΔCt + (1|site)) are summarized in Table 3 and Figure 2. Estimated slopes (*β*_1_) ranged from −0.746 (coagulase-negative *Staphylococcus*) to −0.922 (*Enterococcus*), with all slopes being highly statistically significant (*p* ≤ 3.87 × 10^−22^). As an example, the *E. coli* model slope was −0.825 (95% CI −0.973 to −0.677), indicating that a 1 unit increase in ΔCt corresponds to a 0.825 log_10_ decrease in CFU (approximately a 6.7-fold reduction in the viable bacterial concentration). Marginal and conditional R^2^ values reported in the table suggest the moderate explanatory power of ΔCt for log_10_CFU in these species. For five of the six species, the mixed models were flagged as singular (near-zero estimated site random-intercept variance), implying negligible between-site variance and thus, stable assay calibration across sites. RMSE (root mean square error) values ranged from 0.31 to 0.46 log_10_CFU, indicating good predictive precision at the per-specimen level. Coagulase-negative *Staphylococcus* was the exception, with a non-singular fit and a slightly higher conditional R^2^ than marginal R^2^, which was consistent with a modest site effect for that organism.

Figure 2 displays specimen-level scatterplots of ΔCt (Ct_target − IC_Ct) vs. log_10_CFU, with the species-specific linear mixed-effects prediction lines for the six taxa that met the matched-pairs inclusion criterion. All species show a clear inverse relationship: Spearman ρ ranged from −0.641 (*Pseudomonas*) to −0.751 (coagulase-negative *Staphylococcus*), and mixed-effects slopes ranged roughly from −0.746 to −0.922 log_10_CFU per ΔCt unit (representing ≈5.6–8.4-fold CFU change per 1-Ct change). For example, the *E. coli* panel (A) shows ρ = −0.654 with a slope ≈ −0.825, indicating that a 1 unit higher ΔCt corresponds to ~0.83 log_10_ fewer CFU. The panels also show modest scatter around the fitted lines, reflecting sample-level variability; nonetheless, the consistency of the slope direction and magnitude across the taxa demonstrates reproducible, species-specific calibration between the normalized PCR signal and viable bacterial burden in this dataset.

#### 3.2.3. Discriminatory Performance (ROC) of ΔCt

**Figure 3:**
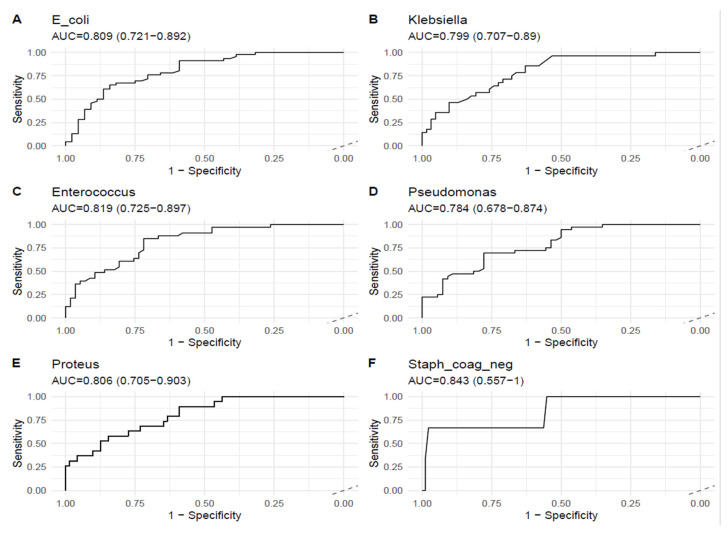
Receiver-operating characteristic (ROC) curves for ΔCt, predicting the culture-defined burden for six uropathogens (**A**–**F**). ROC panels display sensitivity vs. 1—specificity for ΔCt as a predictor of culture burden thresholds; each panel reports the area under the curve (AUC) with the 95% confidence interval: *E. coli* AUC = 0.809 (0.721–0.892), *Klebsiella* AUC = 0.799 (0.707–0.890), *Enterococcus* AUC = 0.819 (0.725–0.897), *Pseudomonas* AUC = 0.784 (0.678–0.874), *Proteus* AUC = 0.806 (0.705–0.903), and coagulase-negative *Staphylococcus* AUC = 0.843 (0.557–1.000).

Receiver-operating characteristic analyses evaluating ΔCt discrimination of the culture-defined burden are shown in Table 4 and Figure 3. For the six species with *N* = 90 each, the AUC point estimates ranged from 0.784 (*Pseudomonas*) to 0.843 (coagulase-negative *Staphylococcus*), indicating the good discriminatory ability of ΔCt in predicting the culture burden. Confidence intervals varied in width between species; notably, the CI for coagulase-negative *Staphylococcus* was wide (0.557–1.000), reflecting greater sample variability for that species in these analyses. ROC computation for several species–threshold combinations at low CFU cut points (T = 1 × 10^3^ and 1 × 10^4^ CFU/mL) was not possible when the binary culture outcome lacked class variation (i.e., when nearly all culture-positive measurements exceeded the threshold); this was a limitation of the available distribution, rather than the analytic method.

Clinical threshold equivalence: ΔCt values between approximately 24 and 26 corresponded to culture burdens of 10^3^–10^5^ CFU/mL, representing the practical diagnostic transition between low-level and clinically significant bacteriuria.

Figure 3 summarizes ROC analyses evaluating ΔCt discrimination of the culture-derived burden for the six species. AUC estimates clustered between 0.78 and 0.84, indicating the good discriminatory performance of the normalized PCR signal for predicting the culture burden in the evaluated taxa. Confidence intervals vary by species (widest for coagulase-negative *Staphylococcus*, reflecting greater sample variability), and several low-threshold ROC computations were precluded in other parts of the dataset by a lack of binary outcome variation. Overall, these ROC results corroborate the calibration analyses: ΔCt provides useful semi-quantitative discrimination of the culture burden, but some misclassification remains at the specimen level, reinforcing the need to interpret ΔCt within the clinical and pre-analytic context.

### 3.3. Sensitivity Analysis for Ct→CFU Correlation, Calibration, and ROC Analyses

The sensitivity set comprised eight species with matched PCR and quantitative culture pairs (Table 5, Table 6 and Table 7). Correlation, mixed-effects regression, and ROC analyses produced internally consistent results: ΔCt was strongly and inversely associated with culture recovery across species, the mixed models yielded robust negative ΔCt slopes with bootstrap CIs that excluded zero for all species, and ΔCt showed a good discriminatory performance for the elevated culture burden (AUC range ≈ 0.74–0.94). These sensitivity results are concordant with the main analyses and reinforce the conclusion that a stronger PCR signal (lower ΔCt) corresponds to a higher viable bacterial load.

Correlation results (Table 5): Spearman and Pearson correlations between ΔCt and log_10_CFU were uniformly negative and statistically highly significant across species, indicating both monotonic and approximately linear inverse relationships: correlations are large in magnitude (absolute ρ and r typically >0.5), which is consistent with a strong inverse relationship between ΔCt and culture burden across diverse uropathogens.

Mixed-effects regression (Table 6)*:* Mixed models adjusted for IC_Ct, the collection method, and prior antibiotics produced consistently negative ΔCt slopes (α_1_) with narrow bootstrap percentile 95% CIs (bootMer, nsim = 2000); for each 1 unit decrease in ΔCt (i.e., stronger PCR signal), the models predict an increase in log_10_CFU of approximately the absolute value of the slope (e.g., ~0.38 log_10_ for *E. coli*, roughly a 2.4-fold increase). All bootstrap CIs exclude zero, indicating robust associations in this sensitivity set.

**Figure 4:**
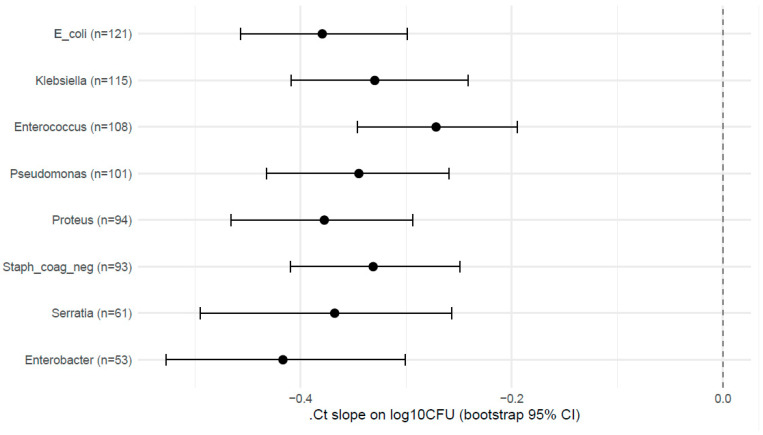
Bootstrap forest plot of ΔCt→log_10_CFU slopes in the sensitivity set (*n* ≥ 50). Each point represents the estimated slope (ΔCt coefficient, α_1_) from the species-specific mixed-effects regression model, with horizontal lines showing the 95% bootstrap confidence intervals (2000 replicates). Negative slopes indicate an inverse relationship between normalized molecular signal (ΔCt = Ct_target − IC_Ct) and viable bacterial burden, quantified by culture. Estimates are presented for eight uropathogens (*E. coli*, *Klebsiella* spp., *Enterococcus* spp., *Pseudomonas* spp., *Proteus* spp., coagulase-negative *Staphylococcus*, *Serratia* spp., and *Enterobacter* spp.). All slopes were significantly negative (*p* < 0.001), which is consistent with the primary analyses (*N* ≥ 90) and confirms that stronger molecular signal corresponds to higher culture-quantified bacterial loads across the taxa.

Discriminatory performance (ROC; Table 7, Figure 4)*:* Using log_10_CFU ≥ 5.0 to define the high burden, ΔCt discriminated the high vs. lower culture burden with consistently good AUCs; across species, ΔCt demonstrated good discrimination for the chosen high-burden threshold (AUCs generally >0.75). Youden cut points and operating characteristics varied by species; prevalence in these sensitivity strata was high (many samples exceeded the 5.0 cutpoint), which increased the AUC stability but also limited generalizability to lower-prevalence settings.

Synthesis and analysis consistency: The sensitivity results (Table 5, Table 6 and Table 7) are internally consistent and concordant with the primary analyses (which used other prespecified sample rules): (1) ΔCt is strongly inversely correlated with CFU across multiple uropathogens; (2) mixed models adjusted for IC_Ct and pre-analytic covariates produce negative ΔCt slopes with bootstrap Cis, excluding zero; (3) ΔCt shows useful discrimination for high bacterial burden (AUCs mostly >0.8). These sensitivity findings (including *Proteus* and additional *Enterobacterales*) strengthen the primary inference that the quantitative PCR signal provides meaningful information about the viable bacterial burden, while underscoring species-specific thresholds and the need to report uncertainty (bootstrap CIs) when operationalizing cut points.

These sensitivity analyses (*n* ≥ 50 strata) corroborate and extend the primary Ct→CFU calibration: the quantitative PCR signal (ΔCt) reliably tracks the viable bacterial burden across multiple common urinary pathogens, including *Proteus* spp. and additional *Enterobacterales*. Species-specific cut points and bootstrap CIs are necessary to reflect remaining uncertainty and to guide any clinical translation.

### 3.4. Pooled Multilevel Analysis of Pre-Analytic Drivers

A pooled multilevel model incorporating the collection method and processing delay (log_10_CFU ~ ΔCt × collection_method + ΔCt × time_to_processing_h + (1|site) + (1|run)) is summarized in Table 8. In this pooled analysis, ΔCt retained a strong, highly significant inverse association with log_10_CFU (estimate −0.8852; SE 0.0528; t = −16.765; *p* < 0.001). The collection method (catheter vs. clean-catch) had a small, non-significant main effect (estimate 0.0301; *p* > 0.05), and the ΔCt × collection_method interaction was likewise not significant (estimate 0.0065; *p* > 0.05), indicating no measurable effect of the collection technique on the ΔCt-CFU calibration after adjustment. By contrast, time to processing (hours) showed a statistically significant negative association with recovered CFU (estimate −0.0484 per hour; 95% CI −0.086 to −0.011; *p* < 0.05), and the ΔCt × time_to_processing_h interaction was also statistically significant (estimate −0.0188; 95% CI −0.032 to −0.005; *p* < 0.05). These estimates indicate that processing delays are associated with modest but measurable declines in culture recovery and that the ΔCt→log_10_CFU relationship becomes more negative with longer delays (i.e., for a given ΔCt, the culture-recovered CFU is lower when processing is delayed). The random-effect variance attributable to the run was small but non-zero, while the site random intercept variance was negligible; most observed variability remained at the sample level.

**Figure 5:**
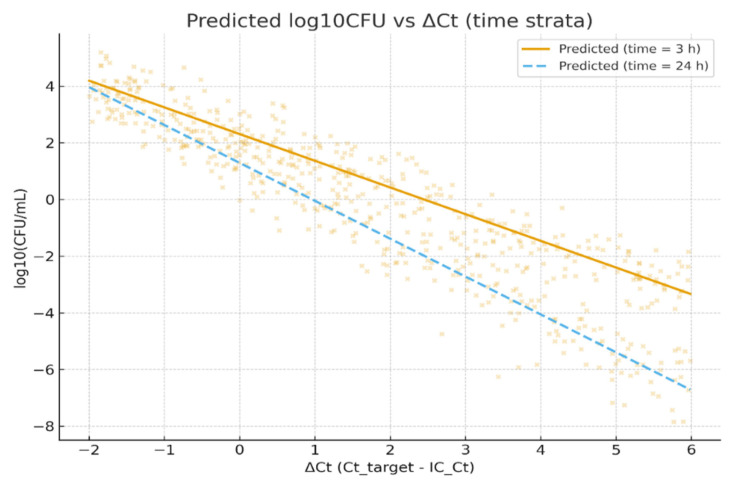
Predicted Effect of Processing Delay on ΔCt-CFU Relationship. This figure shows the observed vs. predicted log_10_CFU as a function of ΔCt, illustrating the effect of a specimen-processing delay. Points show observed data; solid line = model prediction at 3 h turnaround; dashed line = prediction at 24 h turnaround. Predictions are derived from the pooled multilevel model (log_10_CFU ~ ΔCt × time_to_processing + ΔCt × collection_method + (1|site) + (1|run)), holding other covariates constant. Longer delays are associated with systematically lower predicted culture recovery for the same ΔCt, consistent with the loss of bacterial viability over time.

Figure 5 illustrates the modeled relationship between the normalized PCR signal (ΔCt) and viable bacterial burden (log_10_CFU), using the pooled multilevel model described in Section 2.5. The scatterplot displays observed log_10_CFU values (points) plotted against ΔCt, overlaid with two predicted regression lines representing short (3 h; solid line) and long (24 h; dashed line) processing delays, holding other covariates constant (collection method = clean-catch, mean IC_Ct).

The two prediction lines highlight the interaction between ΔCt and time-to-processing: for a given ΔCt, specimens processed after 24 h yield lower predicted culture recovery compared with those processed within 3 h. This visualization supports the model finding that longer processing times modestly attenuate culture-detected viable counts, increasing the likelihood of PCR-positive/culture-negative discordance. The figure therefore contextualizes how a pre-analytic delay can influence the quantitative agreement between molecular and culture-based detection methods.

### 3.5. Summary of Quantitative Results

Across all primary analyses (species with *N* ≥ 90), ΔCt (Ct_target normalized to IC_Ct) demonstrated a strong and statistically significant inverse correlation with quantitative culture (log_10_CFU), confirming that lower ΔCt values (representing stronger molecular signal) correspond to higher viable bacterial loads. Species-specific calibration slopes ranged from −0.75 to −0.92 log_10_CFU per 1 unit ΔCt, equivalent to approximately 5.6–8.4-fold changes in viable burden per Ct unit. This relationship was consistent across uropathogens and across collection sites, with negligible random site variance indicating that inter-site technical variability was minimal.

Discriminatory (ROC) analyses further supported the semi-quantitative validity of ΔCt, with AUCs ranging from 0.78 to 0.84 for the primary set of six taxa, and from 0.75 to 0.94 in the expanded sensitivity set (*n* ≥ 50, eight species). Across both analyses, Youden-derived ΔCt cutpoints around 24–26 optimally discriminated culture-defined high-burden bacteriuria (≥10^5^ CFU/mL), demonstrating clinically interpretable “threshold equivalence” between molecular and culture quantification.

Pre-analytic effects were minor but measurable. The collection method (catheter vs. clean-catch) did not significantly alter the ΔCt-CFU calibration, while time-to-processing significantly reduced the recovered CFU and intensified the negative ΔCt-CFU slope, indicating that longer delays promote partial loss of culturability and contribute to PCR^+^/culture^−^ discordance. Figure 4 illustrates this effect, showing a lower predicted CFU for identical ΔCt values when processing was delayed from 3 to 24 h.

Sensitivity analyses (including (i) expansion to species with *n* ≥ 50 matched pairs and (ii) exclusion of non-detects) yielded a consistent direction and magnitude of associations, reinforcing the robustness of the ΔCt calibration and discrimination models. Collectively, these findings demonstrate that ΔCt provides a reproducible, semi-quantitative indicator of bacterial load across uropathogens, which is resilient to modest pre-analytic variation and suitable for stewardship-integrated diagnostic use.

## 4. Discussion

In this ad hoc analysis of 1027 paired multiplex-PCR and quantitative urine culture specimens from the NCT06996301 trial, normalized PCR signal (ΔCt = Ct_target − IC_Ct) demonstrated a strong, consistent inverse relationship with the culture-quantified bacterial burden across six common uropathogens. Species-specific mixed-effects slopes clustered between approximately −0.75 and −0.92 log_10_CFU per ΔCt unit, and ROC AUCs for the ΔCt discrimination of the culture burden were generally in the 0.78–0.84 range, where computation was possible. Across primary and sensitivity strata, the mixed-model slopes indicate that each 1 unit change in ΔCt corresponds to a clinically meaningful change in log_10_CFU (sensitivity analyses produced species-dependent slope estimates in the ≈−0.3 to −0.9 range), and bootstrap percentile CIs confirm that several of these slopes remain robust against small-sample uncertainty. Observed slopes represent meaningful effect sizes that are both biologically plausible and operationally useful. Overall, ROC AUCs for ΔCt discrimination of high culture burden were consistently in the moderate-to-good range (≈0.75–0.94 across species and sensitivity sets), supporting the semi-quantitative utility of ΔCt and CU within the operational conditions of this trial. A similar observation was made by Upadhyay et al. [7], where a clear agreement between the PCR-generated Ct/Cq value and the traditional CFU/mL value was generated via microbial culture.

The PCR assay consistently detected more positives than culture across all sites, with a larger PCR^+^/culture^−^ fraction (≈24%) than PCR^−^/culture^+^ (≈6%). This pattern mirrors prior observations that molecular methods detect organisms that culture may miss: particularly, fastidious or slow-growing species outside standard culture detection limits [13,14,15]. Moreover, PCR^+^/culture^−^ discordance increased with longer processing delays, which was a pattern consistent with time-dependent viability loss rather than analytical artifacts, supporting the interpretation that a DNA signal often persists after the loss of cultivability. Classic work and subsequent reviews have documented the PCR detection of nonviable organisms or residual DNA following antimicrobial exposure, reinforcing the need for contextual interpretation of such results. Consequently, PCR^+^/culture^−^ findings should be interpreted in light of the clinical presentation, recent antibiotic use, and specimen quality, and (where appropriate) addressed with adjunctive methods such as viability PCR, repeat culture, or reflex enrichment to distinguish true infection from low-level carriage or remnant DNA [15,16,17]. Importantly, the collection method did not significantly modify the ΔCt-CFU calibration, indicating robustness of the observed molecular–culture relationship under standardized workflows.

When compared with prior work on rapid molecular diagnostics and stewardship [3,4,5,6,7,8], our observed AUC range is consistent with published reports that molecular quantity measures add useful but imperfect discrimination. This places our results within the expected performance envelope for semi-quantitative PCR: ΔCt improves probability estimation of the high culture burden. Rapid molecular testing studies have repeatedly demonstrated that earlier organism/marker detection shortens time-to-effective therapy and helps stewardship teams act sooner, but that molecular signal rarely attains perfect discrimination vs. culture or phenotypic AST [15,16]. In our parent trial’s context (Figure 1), for example, PCR guidance significantly shortened the mean turnaround time (≈49 h vs. ≈104 h) and significantly improved the patients’ favorable clinical outcome (≈88% vs. ≈78%), providing a real-world illustration of how faster molecular results can translate to earlier and more appropriate therapy when acted upon [12,13,14]. A subset of DOC Lab UTM 2.0 targets did not reach the pre-specified matched-pairs threshold for Ct↔CFU calibration; most of these targets are known fastidious uropathogens (e.g., *Actinotignum schaalii*, *Aerococcus* spp., select Gardnerella/other emerging taxa) that are frequently under-detected by routine urine culture [16]. Importantly, when the culture recovered these organisms, the molecular calls were concordant in most paired samples, suggesting PCR is identifying true organisms rather than producing artifactual positives. This pattern argues that PCR expands sensitivity for hard-to-culture taxa but also amplifies the need for cautious interpretation and, where clinically relevant, confirmatory culture or targeted enrichment methods [16,17].

Species-level variation in the ΔCt→CFU slope plausibly reflects multiple biological and pre-analytic factors [17,18,19]. These include (1) genome or plasmid copy number differences (a higher copy number of target genes inflates the PCR signal per viable cell), (2) variations in cell-wall structure affecting DNA extraction efficiency across the taxa, (3) differences in the ratio of extracellular to intracellular DNA (for instance, after antibiotic exposure or partial lysis), (4) heterogeneous within-sample bacterial populations (e.g., resistant subclones or subpopulations in a stationary phase), and (5) species-dependent primer/probe-binding efficiencies. These mechanisms explain why slopes vary across taxa and underscore the critical need for assay- and species-specific calibration, rather than universal ΔCt cutoffs.

Pre-analytic factors are a recognized driver of urine culture variability and of culture vs. molecular discordance [18,19]. In our pooled multilevel model, time from collection to processing was associated with a statistically significant decline in recovered CFU (≈0.048 log_10_CFU per hour, ≈10% reduction per hour) and a significant ΔCt × time_to_processing interaction. This interaction means that for identical ΔCt values, culture recovery is lower when processing is delayed, which is consistent with time-dependent viability loss. These results align with prior studies showing that delayed processing or suboptimal transport conditions alter culture yields and that preservatives and refrigeration have imperfect but often helpful preservative effects; hence, laboratories should define the maximum acceptable time-to-processing windows (for example, ≤12 h without preservative, ≤24 h with validated preservative) and record time-to-processing for interpretive reporting [17,18,19,20].

In our dataset, ΔCt achieved AUCs between ~0.78 and ~0.84: values commonly interpreted as “good discrimination” in diagnostic accuracy studies [21]. Practically, an AUC ≈0.80 means that ΔCt substantially improves the clinician’s post-test probability estimate of the high culture burden, relative to chance; however, specimen-level misclassification remains, so ΔCt is best used to triage patients (early stewardship review, reflex testing), rather than as a sole determinant of treatment changes. We therefore recommend conservative operational use of ΔCt thresholds (see checklist below).

Similar AUC ranges have been reported in other multiplex PCR studies for urinary and respiratory pathogens [7,10], confirming that ΔCt offers discrimination that is consistent with prior work. Our analysis extends prior reports by providing species-specific slope calibrations, bootstrap CIs to reflect small-sample uncertainty, and sensitivity analyses (*n* ≥ 50) that show directional consistency across a broader set of taxa (including *Proteus*).

In contrast, the collection method (catheter vs. clean catch) did not materially change the ΔCt→CFU calibration in the adjusted analyses, suggesting that, within this dataset and its operational SOPs, the relationship between the normalized PCR signal and culture burden is robust against the specimen collection modality. However, one site (Norman) had a disproportionately high proportion of catheter samples (≈80%); this concentration could confound site-level comparisons and therefore warrants caution in interpreting any single-site differences, despite the pooled model’s null interaction. The collection technique can still affect contamination rates’ interpretation and therefore should remain a required report element in operational implementations [19,20,21].

The strong species-specific calibration slopes we observed support the practical feasibility of reporting semi-quantitative PCR results that are mapped to culture thresholds (e.g., categories approximating ≥10^3^, ≥10^4^, ≥10^5^ CFU/mL). Nevertheless, important caveats remain; model singularity (near-zero estimated site variance) suggests negligible between-site variance under our harmonized SOPs, but may also reflect limited site heterogeneity and therefore does not guarantee transportability to different laboratory networks. Class imbalance at lower CFU thresholds limited ROC analyses for T = 10^3^ and 10^4^ CFU/mL in some species, so discrimination at very low burdens is less well-characterized in this cohort.

Operationally, our results suggest several pragmatic recommendations for laboratories and clinical programs, considering the implementation of semi-quantitative multiplex PCR for cUTI management [17,18,19,20,21]. Laboratories should (1) derive assay- and species-specific ΔCt↔CFU calibration curves within their exact operational workflows (including extraction methods, instrument platforms, and internal controls), rather than relying on the manufacturer or external thresholds alone; (2) include per-sample internal control Ct and time-to-processing metadata with molecular reports so clinicians can interpret PCR^+^/culture^−^ results in context; and (3) prioritize specimen transport times, validated preservatives (where appropriate), and temperature control to preserve culture viability when AST is needed. These recommendations reflect both the observed empirical calibration and the pre-analytic sensitivity of the culture [22,23,24,25,26,27].

Building upon these findings, we propose an operational checklist to facilitate standardized implementation and quality assurance:

1. Derive local, assay- and species-specific calibration curves, using paired PCR and quantitative culture data from the laboratory’s own workflows (same extraction, instrument, run SOPs, and internal control). Avoid direct transfer of ΔCt cutpoints across platforms without internal validation.
2. Report ΔCt with interpretive comments and internal-control acceptance ranges (e.g., IC_Ct 23–27). Example: “ΔCt = X (semi-quantitative): consistent with high culture burden (≥10^5^ CFU/mL) for *E. coli* in this laboratory; confirmatory culture and AST pending.”
3. Include pre-analytic metadata (time-to-processing and collection method) or an automated QC flag when time-to-processing exceeds the validated window (e.g., >12 h). Our pooled model indicates longer processing times, lower CFU recovery, and increased ΔCt-CFU discordance.
4. Use conservative ΔCt thresholds for autonomous clinical actions, employing them to trigger an early stewardship review or reflex confirmatory testing, rather than direct therapy changes.
5. Validate ΔCt thresholds prospectively in an independent cohort before clinical deployment.
6. Integrate ΔCt QC dashboards tracking IC_Ct distributions, inhibition rates, and time-to-processing metrics to maintain analytic fidelity over time.

Together, these recommendations operationalize the observed calibration behavior, providing a roadmap for laboratories to implement ΔCt-based semi-quantitative reporting within quality-assured, stewardship-aware workflows.

A simple decision sketch suggests that modest ΔCt discrimination (AUC ≈ 0.8) can be clinically valuable when combined with high-PPV markers and when time gains from PCR meaningfully increase the probability of receiving effective empiric therapy (as we observed). From an operational viewpoint, the highest value of semi-quantitative reporting is in settings where earlier therapy materially changes outcomes, and when the culture is retained in parallel to provide definitive MICs and surveillance isolates.

Strengths of this analysis include the large number of paired PCR and quantitative culture specimens, the inclusion of a per-sample internal control that enabled ΔCt normalization, and detailed pre-analytic metadata permitting explicit modeling of time-to-processing and collection method effects. Limitations include single-assay dependence (DOC Lab UTM 2.0), limiting the transferability of numeric cutpoints; the paucity of low-burden specimens that reduce precision for low CFU thresholds (relevant to CAUTI and early infections); and incomplete metadata on transport temperature and preservative use, a major pre-analytic confounder that could partly explain the time-to-processing effect. Model singularity implies small between-site variance in our harmonized network but does not obviate the need for external validation. ΔCt remains susceptible to assay- and workflow-specific biases; local calibration and reflex culture remain essential.

Importantly, our results support the feasibility of deriving useful ΔCt cutpoints that map to the culture %CFU and provide the analytical foundation for interpreting the clinical outcomes observed in the parent randomized trial, NCT06996301. While this ad hoc analysis focuses on analytical calibration and diagnostic performance, the parent trial independently demonstrated improved patient-level outcomes (PCR-guided management: 88% favorable clinical response vs. 78% under conventional culture-based management) and enhanced antimicrobial stewardship efficiency [12,13,14]. Thus, the current findings should be viewed as complementary, establishing the mechanistic and quantitative link between the ΔCt signal and viable bacterial burden that underpins those observed clinical benefits, rather than as direct evidence of clinical effectiveness [26,27,28].

In summary, the Ct from the DOC Lab UTM 2.0 multiplex PCR panel provides a reproducible semi-quantitative signal that correlates strongly with the culture-measured bacterial burden across multiple uropathogens in the NCT06996301 dataset. A similar observation was made by Upadhyay et al. [7], where a clear agreement between the PCR-generated Ct/Cq value and the traditional CFU/mL value was generated via microbial culture. Time-to-processing materially affects culture recovery and increases PCR^+^/culture^−^ discordance; the collection method had no significant adjusted effect on calibration in this dataset. The translation of these calibration data into routine reporting should be accompanied by explicit internal-control reporting, transparency regarding specimen handling metadata, and prospective validation in independent operational contexts. Methodological refinements that reduce the detection of nonviable DNA and rigorous genotype→phenotype AMR concordance work will be necessary steps before molecular testing can reliably replace culture for all diagnostic and stewardship purposes.

## 5. Conclusions

In this ad hoc paired analysis of multiplex PCR and quantitative urine culture from the NCT06996301 trial, the normalized PCR signal (ΔCt) showed a strong, reproducible inverse correlation with the culture-quantified bacterial burden across multiple common uropathogens and demonstrated good discrimination (AUC ≈ 0.78–0.84) for clinically relevant thresholds where ROC analysis was feasible. These findings, supported by a broader sensitivity analysis (*n* ≥ 50), confirm that ΔCt provides a stable, semi-quantitative molecular proxy for the viable bacterial load.

The PCR assay consistently detected more positives than the culture, with PCR^+^/culture^−^ discordance increasing with the processing delay: a pattern consistent with time-dependent viability loss, rather than the analytical artifact. The collection method did not significantly alter the calibration, indicating the robustness of ΔCt-CFU relationships under standardized workflows.

This study extends prior observations [7] by providing the first species-specific, mixed-effects calibration and ROC analysis linking ΔCt to quantitative CFU across multiple uropathogens, validated through sensitivity analyses. Our results support the development of assay- and species-specific ΔCt cutpoints that approximate key clinical thresholds (10^3^–10^5^ CFU/mL) and the routine inclusion of internal-control Ct and specimen metadata to improve interpretability and quality control.

Clinically, ΔCt from the DOC Lab UTM 2.0 multiplex PCR panel provides a reproducible, analytically valid semi-quantitative measure of bacterial load across multiple uropathogens. The calibration, discriminatory performance, and pre-analytic stability of ΔCt meet the expectations for analytical validity and clinical validity. When integrated within the real-world clinical context demonstrated in the NCT06996301 randomized trial, ΔCt provides actionable information that supports meaningful improvements in clinical management and antimicrobial stewardship. Nonetheless, culture remains essential for phenotypic susceptibility testing. Further prospective, cross-platform validation and viability-enrichment or genotype-to-phenotype concordance studies are needed before molecular semi-quantitation can replace culture in therapeutic workflows for complicated UTIs.

## Figures and Tables

**Table 1:** Site-level QC summary.

Site/POL	*n*_Samples	% Culture_pos	% PCR_pos	% PCR^+^/Culture^−^	% PCR^−^/Culture^+^	Median log_10_CFU	IC Ct Pos Window (min_max)	Median Ct Overall	Median IC_Ct	% Inhibited	% PCR Failures	% Catheter
Augusta	205	68.78	88.87	21.95	5.36	5.02	10–32	26.02	25.97	1.95	1.37	0
Albany	192	66.14	83.85	21.13	4.16	4.96	10–33	24.84	26.22	1.17	1.75	0
Norman	224	69.91	83.92	24.55	6.25	5.15	10–33	24.48	25.51	0.89	1.11	80
Phoenix	157	69.42	89.81	22.29	5.09	5.05	10–31	23.85	25.58	0.64	0.00	0
Silicon Valley	29	65.51	82.76	24.14	6.90	5.63	10–32	22.56	25.48	1.45	0.00	0
Southeastern	220	69.10	88.63	24.09	6.36	4.85	10–33	24.41	25.02	1.27	1.12	0

**Table 2:** Correlation (ΔCt vs. log_10_CFU) by species. Strong and statistically significant negative correlations demonstrate that ΔCt reliably tracks quantitative culture burden across uropathogens.

Species	*n*	Spearman_rho	Spearman_*p*	Pearson_r	Pearson_*p*
*E. coli*	121	−0.654	2.81 × 10^−12^	−0.751	1.45 × 10^−17^
*Klebsiella*	115	−0.653	3.08 × 10^−12^	−0.804	1.31 × 10^−21^
*Enterococcus*	108	−0.71	4.93 × 10^−15^	−0.829	6.4 × 10^−24^
*Pseudomonas*	101	−0.641	9.92 × 10^−12^	−0.756	7.41 × 10^−18^
*Proteus*	94	−0.677	2.43 × 10^−13^	−0.769	8.99 × 10^−19^
*Staph_coag_neg*	93	−0.751	1.46 × 10^−17^	−0.766	1.39 × 10^−18^

**Table 3:** Linear mixed-effects calibration models for ΔCt, predicting log_10_CFU per species. Negative slopes indicate that a higher molecular signal (lower ΔCt) corresponds to higher viable bacterial burden.

Species	Slope	Slope_se	Slope_t	Slope_*p*	Slope_95L	Slope_95U	R^2^_Marginal	R^2^_Conditional	Singular
*E. coli*	−0.825	0.075	−10.956	3.01 × 10^−18^	−0.973	−0.677	0.573	0.573	TRUE
*Klebsiella*	−0.882	0.068	−12.883	3.87 × 10^−22^	−1.016	−0.748	0.654	0.654	TRUE
*Enterococcus*	−0.922	0.066	−14.069	1.89 × 10^−24^	−1.051	−0.794	0.696	0.696	TRUE
*Pseudomonas*	−0.791	0.071	−11.121	1.39 × 10^−18^	−0.93	−0.651	0.583	0.583	TRUE
*Proteus*	−0.901	0.084	−10.722	9.15 × 10^−18^	−1.066	−0.736	0.622	0.622	TRUE
*Staph_coag_neg*	−0.746	0.064	−11.71	2.09 × 10^−19^	−0.871	−0.621	0.582	0.612	FALSE

**Table 4:** ROC performance of ΔCt for predicting culture burden ≥10^5^ CFU/mL. AUC values between 0.78 and 0.84 indicate good diagnostic discrimination.

Species	AUC	AUC_95L	AUC_95U	Youden ΔCt Cut-Point	Sensitivity	Specificity	Prevalence (≥10^5^ CFU/mL)
*E. coli*	0.809	0.721	0.892	24.4	0.81	0.73	0.73
*Klebsiella*	0.799	0.707	0.89	25.5	0.78	0.79	0.79
*Enterococcus*	0.819	0.725	0.897	24.7	0.53	0.93	0.86
*Pseudomonas*	0.784	0.678	0.874	24.9	0.62	0.95	0.81
*Proteus*	0.806	0.705	0.903	24.0	0.66	0.89	0.81
*Staph_coag_neg*	0.843	0.557	1	26.8	0.92	0.68	0.76

**Table 5:** Correlation between ΔCt and log_10_CFU (sensitivity set, species with *n* ≥ 50). Per-species Spearman (ρ) and Pearson (r) correlations between ΔCt and quantitative culture (log_10_CFU). Negative values indicate inverse association (stronger PCR signal/lower ΔCt associated with higher culture burden). *p*-values are two-sided.

Species	*n*	Spearman ρ	*p* (Spearman)	Pearson r	*p* (Pearson)
*E. coli*	121	−0.647	<1 × 10^−16^	−0.663	1.26 × 10^−16^
*Klebsiella* spp.	115	−0.560	<1 × 10^−10^	−0.582	8.77 × 10^−12^
*Enterococcus* spp.	108	−0.575	<1 × 10^−10^	−0.558	3.56 × 10^−10^
*Pseudomonas* spp.	101	−0.565	9.6 × 10^−10^	−0.601	3.01 × 10^−11^
*Proteus* spp.	94	−0.649	<1 × 10^−13^	−0.664	3.08 × 10^−13^
Coagulase-neg. *Staphylococcus*	93	−0.552	1.7 × 10^−8^	−0.619	3.7 × 10^−11^
*Serratia* spp.	61	−0.597	6.7 × 10^−7^	−0.637	3.3 × 10^−8^
*Enterobacter* spp.	53	−0.718	3.6 × 10^−9^	−0.724	9.3 × 10^−10^

Spearman and Pearson correlations quantify the inverse relationship between normalized PCR signal (ΔCt) and quantitative culture (log_10_CFU). All correlations are significant, confirming that increasing ΔCt (weaker molecular signal) corresponds to lower viable bacterial counts.

**Table 6:** Mixed-effects regression of log_10_CFU on ΔCt (sensitivity set) with bootstrap 95% CIs. Each row shows species, sample size (*n*), ΔCt slope estimate (α_1_) from the mixed model log_10_CFU ~ ΔCt + IC_Ct + collection_method + prior_abx + (1|site), approximate Wald-type *p*, and bootstrap percentile 95% confidence interval for α_1_ (bootMer, nsim = 2000). Negative slopes indicate that a lower ΔCt predicts a higher recovered CFU; the CI columns report parametric bootstrap percentiles (or residual bootstrap fallback if indicated).

Species	ΔCt Estimate	SE	t	*p* (Approx.)	95% CI (Lower)	95% CI (Upper)
*E. coli*	−0.379	0.039	−9.67	3.9 × 10^−22^	−0.457	−0.299
*Klebsiella* spp.	−0.330	0.044	−7.54	4.6 × 10^−14^	−0.409	−0.241
*Enterococcus* spp.	−0.272	0.040	−6.86	7.1 × 10^−12^	−0.346	−0.195
*Pseudomonas* spp.	−0.345	0.043	−7.94	2.1 × 10^−15^	−0.432	−0.260
*Proteus* spp.	−0.377	0.044	−8.54	1.3 × 10^−17^	−0.466	−0.293
Coagulase-neg. *Staphylococcus*	−0.331	0.042	−7.86	3.7 × 10^−15^	−0.410	−0.249
*Serratia* spp.	−0.368	0.061	−6.02	1.8 × 10^−9^	−0.495	−0.257
*Enterobacter* spp.	−0.417	0.059	−7.13	1.0 × 10^−12^	−0.527	−0.301

Each model includes fixed effects for ΔCt, internal-control Ct, collection method, and prior antibiotic exposure with random intercepts for site. Negative slopes confirm that higher ΔCt values predict proportionally lower culture recovery. Bootstrap (2000 resamples) 95% CIs are shown.

**Table 7:** ROC discrimination of −ΔCt for high culture burden (log_10_CFU ≥ 5.0). Per-species area under the ROC curve (AUC) with bootstrap percentile 95% CI (nsim = 2000), Youden ΔCt cut point, sensitivity and specificity at the Youden cutoff, and stratum prevalence of high burden. In ROC estimation, we used −ΔCt as the predictor so that larger predictor values corresponded to the greater marker burden; Youden ΔCt is reported on the ΔCt scale (higher = numerically larger Ct difference).

Species	AUC	95% CI (Lower)	95% CI (Upper)	Youden ΔCt Cut-Point	Sensitivity	Specificity	Prevalence (≥10^5^ CFU/mL)
*E. coli*	0.811	0.728	0.891	24.4	0.81	0.73	0.73
*Klebsiella* spp.	0.824	0.731	0.908	25.5	0.78	0.79	0.79
*Enterococcus* spp.	0.745	0.626	0.850	24.7	0.53	0.93	0.86
*Pseudomonas* spp.	0.814	0.710	0.905	24.9	0.62	0.95	0.81
*Proteus* spp.	0.803	0.699	0.887	24.0	0.66	0.89	0.81
Coagulase-neg. *Staphylococcus*	0.806	0.684	0.907	26.8	0.92	0.68	0.76
*Serratia* spp.	0.871	0.739	0.967	25.0	0.73	0.88	0.74
*Enterobacter* spp.	0.942	0.871	0.992	25.7	0.89	1.00	0.83

Receiver-operating characteristic (ROC) analysis of ΔCt vs. culture reference (≥10^5^ CFU/mL). All species showed AUC ≥ 0.74, indicating good to excellent discrimination. Youden-optimized cut-points cluster around ΔCt ≈ 24–26 cycles, consistent with the semi-quantitative thresholds identified in the main analysis.

**Table 8:** Linear mixed-effects modeling examined the relationship between molecular signal and quantitative culture.

Term	Estimate	Std.Error	df	t Value	Pr (>|t|)	95% CI Lower	95% CI Upper
(Intercept)	2.4531	0.1243	498.58	19.735	<0.001	2.209	2.697
ΔCt	−0.8852	0.0528	539.25	−16.765	<0.001	−0.989	−0.782
collection_method_catheter	0.0301	0.173	537.91	0.174	>0.05	−0.309	0.369
time_to_processing_h	−0.0484	0.0191	512.56	−2.534	0.01–0.05	−0.086	−0.011
ΔCt:collection_method_catheter	0.0065	0.0729	536.28	0.089	>0.05	−0.136	0.149
ΔCt:time_to_processing_h	−0.0188	0.00694	537.85	−2.709	0.01–0.05	−0.032	−0.005

## Data Availability

Due to the sensitive nature of the clinical data and patient confidentiality requirements, the datasets used and/or analyzed during the current study are not publicly available. However, they are available from the corresponding author on reasonable request, provided that the request complies with relevant ethical guidelines and data protection regulations.

## Supplementary Materials

The following supporting information can be downloaded at: https://www.mdpi.com/article/10.3390/diagnostics15232959/s1, Supplementary Methods: Culture Report Sample, PCR Report Sample, Urine Culture, UTI Panel_merged.

## Abbreviations

PCR: polymerase chain reaction; Ct: cycle threshold (also Cq, quantification cycle); ΔCt: delta Ct (Ct_target − IC_Ct, i.e., Ct normalized to the internal control); IC_Ct: internal-control cycle threshold; C&S: culture and susceptibility (quantitative urine culture with phenotypic AST); CFU: colony-forming units (per mL); log_10_CFU: base-10 logarithm of CFU/mL; MIC: minimum inhibitory concentration; log_2_MIC: base-2 logarithm of MIC; AST: antimicrobial susceptibility testing; AST_MIC: numeric MIC value; AST_SIR: categorical AST interpretation (S/I/R); PPV: positive predictive value; NPV: negative predictive value; LR+: positive likelihood ratio; LR−: negative likelihood ratio; ROC: receiver-operating characteristic; AUC: area under the ROC curve; CI: confidence interval; SE: standard error; OR: odds ratio; HR: hazard ratio; IQR: interquartile range; REML: restricted (residual) maximum likelihood; lmer/merMod: mixed-effects model fit using lme4::lmer (merMod object); OLS: ordinary least squares regression; bootMer: lme4 bootstrapping procedure for merMod objects; nsim: number of bootstrap replicates; ACME: average causal mediation effect; ADE: average direct effect; Mod-ITT: modified intention-to-treat cohort; EOS: end-of-study; CAUTI: catheter-associated urinary tract infection; cUTI: complicated urinary tract infection; SOP: standard operating procedure; IC_flag: internal-control failure/inhibition flag.
